# Prediction the prognosis of the poisoned patients undergoing hemodialysis using machine learning algorithms

**DOI:** 10.1186/s12911-024-02443-0

**Published:** 2024-02-06

**Authors:** Mitra Rahimi, Mohammad Reza Afrash, Shahin Shadnia, Babak Mostafazadeh, Peyman Erfan Talab Evini, Mohadeseh Sarbaz Bardsiri, Maral Ramezani

**Affiliations:** 1 Toxicological Research Center, Excellence Center & Department of Clinical Toxicology, Loghman Hakim Hospital, Shahid Beheshti University of Medical Sciences, Tehran, Iran; 2 Department of Artificial Intelligence, Smart University of Medical Sciences, Tehran, Iran; 3 Department of Clinical Toxicology, Loghman Hakim Hospital, Shahid Beheshti University of Medical Sciences, Tehran, Iran; 4 Department of Clinical Toxicology, Firouzgar Hospital, Iran University of Medical Sciences, Tehran, Iran; 5 Department of Pharmacology, School of Medicine, Arak University of Medical Sciences, Arak, Iran; 6 Traditional and Complementary Medicine Research Center, Arak University of Medical Sciences, Arak, Iran

**Keywords:** Renal dialysis, Poisoning, Prognosis, Machine learning

## Abstract

**Background:**

Hemodialysis is a life-saving treatment used to eliminate toxins and metabolites from the body during poisoning. Despite its effectiveness, there needs to be more research on this method precisely, with most studies focusing on specific poisoning. This study aims to bridge the existing knowledge gap by developing a machine-learning prediction model for forecasting the prognosis of the poisoned patient undergoing hemodialysis.

**Methods:**

Using a registry database from 2016 to 2022, this study conducted a retrospective cohort study at Loghman Hakim Hospital. First, the relief feature selection algorithm was used to identify the most important variables influencing the prognosis of poisoned patients undergoing hemodialysis. Second, four machine learning algorithms, including extreme gradient boosting (XGBoost), histgradient boosting (HGB), k-nearest neighbors (KNN), and adaptive boosting (AdaBoost), were trained to construct predictive models for predicting the prognosis of poisoned patients undergoing hemodialysis. Finally, the performance of paired feature selection and machine learning (ML) algorithm were evaluated to select the best models using five evaluation metrics including accuracy, sensitivity, specificity the area under the curve (AUC), and f1-score.

**Result:**

The study comprised 980 patients in total. The experimental results showed that ten variables had a significant influence on prognosis outcomes including age, intubation, acidity (PH), previous medical history, bicarbonate (HCO3), Glasgow coma scale (GCS), intensive care unit (ICU) admission, acute kidney injury, and potassium. Out of the four models evaluated, the HGB classifier stood out with superior results on the test dataset. It achieved an impressive mean classification accuracy of 94.8%, a mean specificity of 93.5 a mean sensitivity of 94%, a mean F-score of 89.2%, and a mean receiver operating characteristic (ROC) of 92%.

**Conclusion:**

ML-based predictive models can predict the prognosis of poisoned patients undergoing hemodialysis with high performance. The developed ML models demonstrate valuable potential for providing frontline clinicians with data-driven, evidence-based tools to guide time-sensitive prognosis evaluations and care decisions for poisoned patients in need of hemodialysis. Further large-scale multi-center studies are warranted to validate the efficacy of these models across diverse populations.

## Introduction

Intoxication is described as the appearance of disease symptoms in a human due to exposure to hazardous substances [1]. Research indicates that intoxication is prevalent globally among individuals visiting emergency rooms [2–4]. The World Health Organization (WHO) approximates that each year, 3.5 to 5 million people unintentionally suffer from poisoning worldwide. About three million are severe, resulting in 20,000 yearly deaths [5].

One of the effective methods in treating a poisoned person is to increase excretion. Excretion is the process through which our body eliminates toxins after absorption. Methods include multi-dose activated charcoal, urine alkalization, and extracorporeal treatments [6]. Extracorporeal treatments (ECTRs) are typically administered to a select group of patients who are at risk of experiencing life-threatening toxicity. This specific subset may face prolonged stays in the ICU, where they may require mechanical ventilation and could fall into a coma. Furthermore, these patients are at a high risk of developing permanent disabilities or escalating toxicity, even with supportive care [7]. ECTRs encompass several treatment methods, including hemodialysis (HD), charcoal hemoperfusion (HP), continuous venovenous hemofiltration (CVVH), and continuous venovenous hemodiafiltration (CVVHDF) [8].

Hemodialysis is the best treatment for water-soluble drugs, especially drugs with a molecular weight that can quickly distribute through the smooth membrane. Some examples of this group are salicylates, ethanol, methanol, and lithium [9]. Due to several key advantages, hemodialysis is generally the preferred treatment for most poisonings. It has a lower cost and fewer complications compared to alternatives like hemoperfusion, plasma exchange therapy, and albumin dialysis, and it also excels in treating concurrent metabolic disorders. Additionally, hemodialysis boasts a high clearance capacity for a broad spectrum of foreign substances, further solidifying its role as a reliable and effective treatment option [10]. It is often overlooked that an intoxicated patient can have a significantly altered metabolic profile compared to a patient suffering from renal failure. This is especially true concerning serum levels of potassium, phosphate, and bicarbonate [10].

Previous studies on prognostic factors in hospital hemodialysis patients were based on statistical methods and regression models [11, 12]. A decade-long research conducted at Havana Hospital on patients suffering from end-stage kidney disease revealed certain adverse factors that predict the commencement of hemodialysis. These include conditions like diabetes, hypertension, and chronic anemia. Other factors, including malnutrition, hypoalbuminemia, cardiovascular disease, and liver disease, were added later [13]. These studies do not include poisoned hemodialysis patients, and the studies on the prognostic factors of poisons also include all factors and the outcome, and they have not worked on hemodialysis separately [14–17]. A five-year study on poisoned patients undergoing hemodialysis revealed crucial correlations between patients' outcomes and consciousness level, hypotension, respiratory failure, blood urea nitrogen (BUN), creatinine (Cr), PH, white blood cells (WBC), blood glucose, acute renal failure, and the causes of hemodialysis treatment [18].

Prediction of hemodialysis outcomes depends on many factors and is different in different populations [19]. Risk prediction models are created to estimate the likelihood of an unfavorable outcome, such as mortality, based on various variables including demographic and clinical factors [20, 21]. Traditional techniques like proportional hazards regression (Cox regression) and logistic regression assume a linear relationship between these variables and the outcomes [22]. In recent times, machine learning techniques have emerged as more sophisticated and reliable approaches for predicting outcomes, provided that there is a sufficient amount of data available for analysis. These novel analytical methods have the potential to uncover previously unidentified variables that can enhance the accuracy of predictions [23]. ML refers to the examination and utilization of mathematical algorithms that can enhance their performance autonomously, without the requirement of human involvement. Machine learning algorithms utilize previous data as input and generate new predicted values as output. Machine learning algorithms have been employed in numerous domains to solve a wide range of tasks [24, 25]. For example, in the comparison of death prediction in hemodialysis patients, the logistic regression method and the random forest model have been compared. Baseline data collected at 30 days has demonstrated that random forest is a preferable approach compared to logistic regression in forecasting mortality at 6 months, 1 year, and 2 years, as it yields higher AUCs. Consequently, random forest is the preferred option for developing mortality prediction models in hemodialysis patients [19].

Artificial intelligence is being applied in a variety of medical fields nowadays. Machine learning algorithms are being used to speed up disease diagnosis and guide treatment decisions for a wide range of ailments [25–27]. The utilization of artificial intelligence and machine learning in the field of toxicology, particularly clinical toxicology, is relatively new. Limited research has been conducted on the identification, diagnosis, and treatment of poisoning cases by ML. Hosseini et al. used ML methods to predict the severity of organophosphate poisoning. In their study, the XGBoost model outperformed the other models with an AUC value of 0.907. The three main variables that contributed the most to the prediction of prognosis in patients with organophosphorus poisoning were venous blood gas pH, white blood cells, and plasma cholinesterase activity. XGBoost model accuracy was 90.1%, specificity 91.4%, sensitivity 89.5%, F-measure 91.2% and the kappa statistic was 91.2% [28]. Validation of a machine learning (random forest) predictive model of blood lead levels (EBLLs) and its comparison with simple logistic regression was carried out in the study of Potash et al. The AUC for the random forest was 0.69, while for logistic regression it was 0.64. When identifying the top 5% of children at highest risk for having EBLLs, the random forest model had a positive predictive value of 15.5% and sensitivity of 16.2%, while the logistic regression model had a positive predictive value of 7.8% and sensitivity of 8.1%. Both models had a specificity of over 95%. The machine learning model was surpassed by the regression model in its ability to predict childhood lead poisoning, particularly in identifying children with the greatest risk [29].

Chen et al. used a machine learning approach to identify blood factors to identify the severity of paraquat toxicity and/or diagnose PQ poisoning in patients. The correlation between death from paraquat poisoning and two key factors, WBC and the absolute value of neutrophilic granulocyte (AVNG), was found to be significant. These two factors were determined to be more effective than any other blood index in detecting Paraquat poisoning toxicity at an early stage [30].

As established, patients with poisoning need hemodialysis to face high rates of adverse outcomes. though validated predictive models tailored to this group are lacking. developing ML algorithms to predict critical outcomes, and address a substantial unmet need for evidence-based decision support. In this study, we were looking for the best machine learning model to predict the prognosis hemodialysis in the treatment of poisoned patients.

## Material and methods

### Study roadmap and experiment environment

Based on a single-center registry database, the current retrospective cohort study was conducted at Loghman Hakim Hospital from July 24, 2016, to June 17, 2022. The purpose of the study was to develop a clinical decision support system (CDSS) to forecast the outcomes of dialysis candidates who have been poisoned. This study tested several ML algorithms based on selected variables to predict the prognosis of poisoned patients undergoing hemodialysis. These algorithms' performance was assessed by focusing on the crucial factors determined through a feature selection method. The current study uses four ML algorithms to build prediction models: XGBoost, HGB, K-NN, and AdaBoost classifier. The performance evaluation and algorithm validation criteria were computed. The model development process is structured into four distinct phases. These phases encapsulate the essential steps required for building efficient models. Here is the breakdown:

1. Data Preprocessing
2. Feature Selection and Cross-Validation
3. Execution of Prediction Models
4. Performance Evaluations

**Fig. 1:**
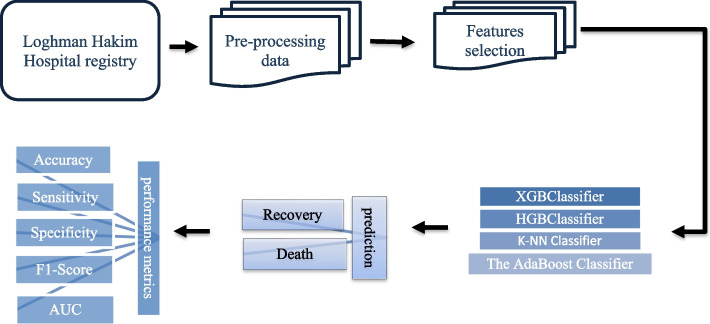
Proposed system roadmap for prognosis of dialysis candidates with poisoning

The study's roadmap for predicting the outcomes of dialysis candidates who have been poisoned is illustrated in Fig. 1. The researchers conducted all algorithmic experiments using Python programming language, including preprocessing, training, and performance evaluation (version 3.7.7).

### Data set description

The study utilized a dataset from the database registry at Loghman Hakim Hospital (Sabara and Shafa centers), affiliated with Shahid Beheshti University of Medical Sciences. The study design received approval from the Ethics Committee at Shahid Beheshti University of Medical Sciences (Ethics Code: IR.SBMU.RETECH.REC.1401.767). Data collection occurred from July 24, 2016, to June 17, 2022. Of 68,181 hospitalized patients from 2016 to 2022 in the poisoning department of Loghman Hakim Hospital in Tehran, 980 patients have been treated using extracorporeal methods (hemodialysis). Among all 980 patients who participated in this study, 418 died, while 562 remained healthy. The research examined a range of variables, including age, sex, type of poisoning, history of underlying disease, medication use and habits, number of times the extracorporeal method was used, type of extracorporeal method, laboratory tests result, and vital signs and patient's outcome. The dependent variable, which may be considered as the outcome of the patients undergoing hemodialysis, was (one feature, death or health), while the analyzed dataset contained descriptive information about responders (55 attributes) (Table 1).

**Table 1:** Demographic, laboratory, and clinical data of patients

Demographic Data			
Variables		Frequency (%)	Values
Gender	Female	187 (19.1%)	Female/ male
	Male	793 (80.9%)	
Age	Under 20 years old	100 (10/4%)	Numerical
	21–40 years	604 (61.6%)	
	41–60 years	211 (21.5%)	
	61–80 years	60 (6.1%)	
	Above 81 years	5 (0.5%)	
Co-ingestion		62 (6.3%)	Yes /No
Smoking		46 (4.7%)	Yes /No
Alcohol consumption		627 (64%)	Yes /No
Opium abuse		75 (7.7%)	Yes /No
Stimulants abuse		13 (1.3%)	Yes /No
History of pervious disease		150 (15.3%)	Yes /No
History of taking medication		77 (7.9%)	Yes /No
Hemoperfusion		9 (0/9%)	Yes /No
Intubation		203 (20.7%)	Yes /No
ICU admission		187 (19.1%)	Yes /No
Antidote therapy		904 (92.2%)	Yes /No
Duration of hospitalization. median (Minimum–Maximum)		2 (1–116)	Numerical
Laboratory and Clinical Data			
13 ≤ GCS < 15		659 (67.2%)	Numerical
8 ≤ GCS < 13		62 (6.3%)	Numerical
Coma (GCS < 8)		108 (11%)	Yes/No
Bradypnea		46 (4.7%)	Numerical
Temperature (Mean ± SD)		36.9 ± 0.56	Numerical
Bradycardia		14 (1.4%)	Numerical
Tachycardia		145 (14.8%)	Numerical
Hypotension		38 (3.9%)	Numerical
Hypertension		293 (29.9%)	Numerical
Metabolic Acidosis		823 (84%)	Numerical
Acute Kidney Injury		536 (54.7%)	Numerical
Renal disease		641(65%)	Yes/No
HCO_3_ (Mean ± SD)		14.4 ± 23.8	Numerical
BUN (meq/l) (Mean ± SD)		36.7 ± 27.2	Numerical
Creatinine (meq/l) (Mean ± SD)		1.7 ± 4.1	Numerical
Blood Glucose (mg/dl) (Mean ± SD)		132.5 ± 66.6	Numerical
Blood PH (Mean ± SD)		7.2 ± 0.4	Numerical
Sodium (meq/l) (Mean ± SD)		137.8 ± 10.8	Numerical
Potassium (meq/l) (Mean ± SD)		4.7 ± 3.4	Numerical
PCO2, PO2, HCO3, BE, Bs, WBC, Hb, Hct, PLT, AST, ALT, ALP, LDH, CPK, CPK mb, PT, PTT, INR^a^		-------	Numerical
Patient Outcomes	Death	418	Yes /No
	Healthy (recovery)	562	Yes /No

^a^PCO2 (partial pressure of carbon dioxide), *PO2* partial pressure of oxygen, *BE* (Base excess), PLT platelet cells, *HGB* hemoglobin, *HCT* hematocrit, *INR* international normalized ratio, *BS* Blood sugar, *LDH* Lactate dehydrogenase, *AST* Aspartate aminotransferase, *ALT* Alanine transaminase, *ALP* Alkaline phosphatase, *CPK* Creatine phosphokinase, *CK-MB* Creatine kinase-MB, *PT* Prothrombin time, *PTT* Partial thromboplastin time

### Outcome variable

This study employs a binary categorical outcome variable to predict prognosis in poisoned patients undergoing hemodialysis. The outcomes are defined as either healthy hospital discharge (indicating sufficient recovery and improvement) or death during hospitalization, which provide clinically meaningful classifications of potential prognoses. This prognostic classification serves as a useful outcome measure for predictive modeling that aims to enhance prognosis evaluation in this patient population.

### Data preprocessing

Preprocessing is pivotal in optimizing data use and training predictive algorithms effectively. This study used several preprocessing techniques to enhance data for predicting dialysis candidates among poisoned patients. These techniques included handling missing values, standard scaling, and min–max scaling. Standard scaling ensures that each feature has a mean of zero and a variance of one, thus aligning all features to a consistent scale. In the process of minimum and maximum scaling, values are adjusted so that all attributes uniformly range from 0 to 1.

Moreover, rows with a significant proportion of missing values, specifically more than 70%, are eliminated. A collaborative effort was made by two authors along with a pair of infectious disease experts. They aimed to identify and rectify noisy and abnormal values, errors, duplicates, and unrelated data.

### Feature selection

Before applying ML algorithms, it is essential to perform feature selection. This is because irrelevant features can seriously undermine the performance of prediction models. By selecting relevant features, the accuracy of prediction models can be improved, and the computational complexity of these models can be reduced. This study utilized the Relief Feature Selection Algorithm to pinpoint the most significant factors in forecasting the prognosis for patients suffering from poisoning and requiring dialysis. The relief algorithm assigns a weight to each feature in the dataset, with the capability of updating these weights as time progresses.

### Model development and evaluation

This research employed a range of ML methods to forecast the outcomes for patients suffering from poisoning and potentially requiring dialysis. The techniques employed include XGB, K-NN, the AdaBoost, and HGBoosting Classifiers as follow.

The "XGB Classifier" refers to the extreme gradient boosting algorithm, which is an ML model used for classification tasks. It is renowned for its effectiveness, speed, and strong predictive performance and is built on gradient-boosting architecture [31].

The k-nearest neighbor classifier is a simple supervised machine learning algorithm that categorizes objects based on the majority class of its k-closest training examples in the feature space. The positive integer k is chosen to optimize classification accuracy on the dataset; small values of k are typically the most effective [32].

AdaBoost is an ensemble learning algorithm that combines multiple weak learners into a single strong classifier through an iterative process. It sequentially trains base models on reweighted training sets to focus more on previously misclassified examples. The base models then vote on the classification output, with more weight given to the stronger learners, resulting in an ensemble that outperforms the individual constituents [33].

Histgradient boosting is an ensemble technique that sequentially trains decision trees on residuals using histogram-based splits for high dimensionality and sparsity. It aggregates iterative predictions from weaker learners into a stronger classifier, formulated as:

$$\mathrm{y }= \sum \mathrm{ Treei}\left({\text{x}}\right)$$

Where y is the final prediction and Treei(x) is the prediction from the decision tree. This gradient-boosting approach enables rapid and effective modeling for sparse, high-dimensional data [34].

For the development and evaluation of the machine learning models, first, the dataset was randomly divided into 90% for training and 10% for testing the models. Second, a tenfold cross-validation technique was used to train and test the ML models over the selected features. Cross-validation helps estimate model performance on unseen data and prevent overfitting. Dividing the data into training and testing sets helps reduce sampling bias and ensures representative distribution between the partitions. This allows for the fair evaluation of model performance on new data.

Cross-validation (CV) helps tune hyperparameters to optimal values for a given dataset while avoiding overfitting. Randomized search cross-validation method was implemented for hyperparameters tuning to identify optimal model architectures.

The performance of Four ML models was assessed using five common evaluation metrics: accuracy, precision, recall, F1-score, and AUC-ROC. These metrics quantify predictive ability on unseen data and suitability for clinical implementation. These metrics were measured using Eqs. 1 to 4. Furthermore, to facilitate a more comprehensive comparison of algorithm performance, additional assessments were conducted, considering the time required to construct the model, and kappa statistic (KS).

1 $$\mathrm{classification \,accuracy}=\frac{\mathrm{true \,positive }({\text{TP}})+\mathrm{true \,negative }({\text{TN}})}{{\text{TP}}+{\text{TN}}+\mathrm{false \,positive }({\text{FP}})+\mathrm{false \,negative }({\text{FN}})}*100$$

2 $$\mathrm{classification \,sensitivity}=\frac{{\text{Tp}}}{{\text{TP}}+{\text{FN}}}*100$$

3 $$\mathrm{classification \,specificity }=\frac{{\text{TN}}}{{\text{TN}}+{\text{FP}}}*100$$

4 $$\mathrm{classification \,error }=\frac{\mathrm{FP }+\mathrm{ FN}}{\mathrm{TP }+\mathrm{ TN }+\mathrm{ FP }+\mathrm{ FN}}*100$$

## Results

### Characteristics of patients

**Fig. 2:**
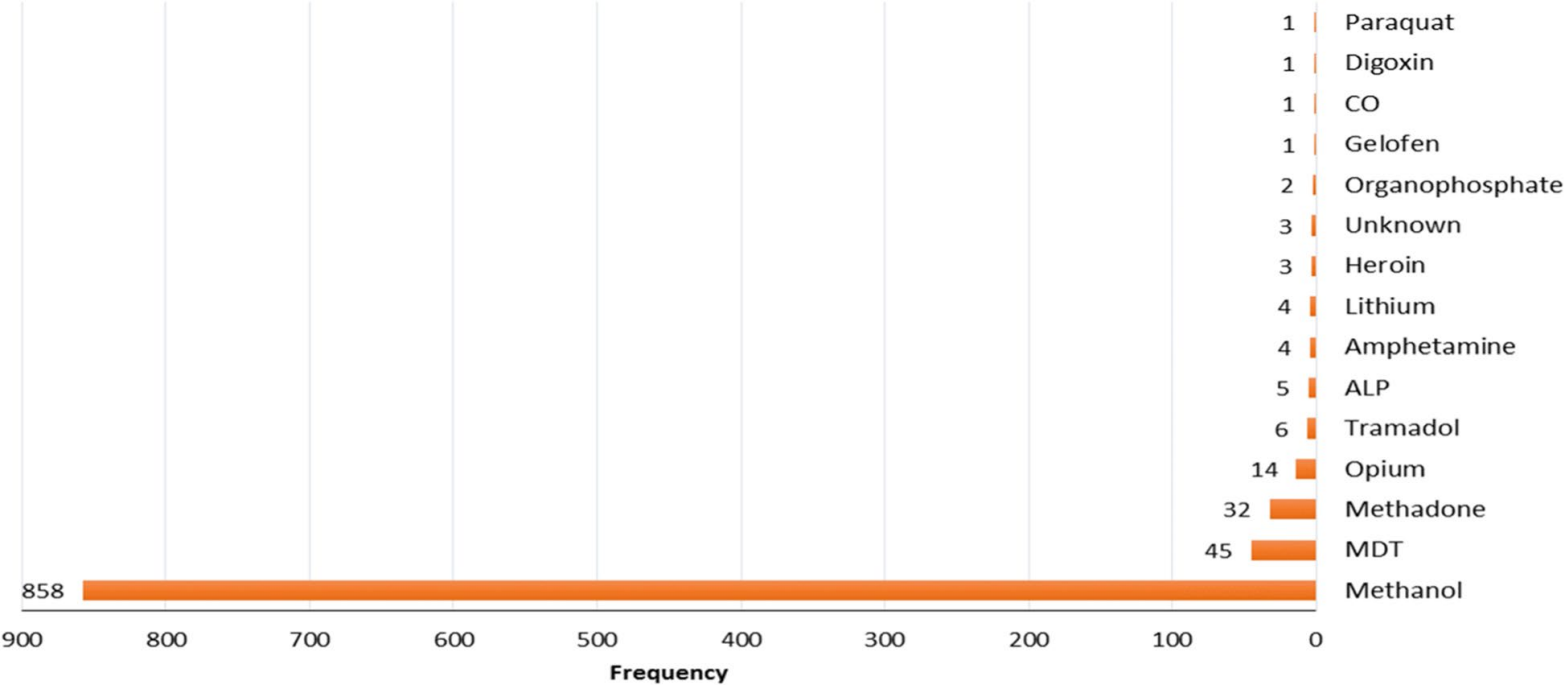
Cause of intoxication in the studied patients. MDT = Multiple Drug Toxicity

Following the implementation of the exclusion criteria, out of the 68,181 patients admitted to the poisoning wards, 980 patients, representing 1.4% of the cases, underwent hemodialysis. Demographic investigation revealed 793 (80.9%) males and 187 (19.1%) females. Detailed demographic data is presented in Table 1. The average age of the subjects was 36.5 years, with a standard deviation of 14 years. The age distribution was significantly different (*p* < 0.001). Six hundred four cases (61.6%) were in the age range of 21–40 years. One hundred seventeen cases (11.9%) had intentional poisoning. As shown in Fig. 2, the highest cause of poisoning was due to methanol consumption (858 cases, 87.6%). Eight hundred thirty cases (84.7%) had no previous history of the disease. Two cases had a history of kidney disease. Nine hundred-three cases (92.1%) had no history of taking drugs, and 627 (64%) had a history of alcohol consumption*.* Hemodialysis was the most widely used extracorporeal method (971 cases, 99.1%). The median number of hemodialysis was one, and the maximum was 18 times. Hemoperfusion was performed for five cases of methanol, three cases of multidrug, and one case of methadone poisoning.

### Feature selection algorithm results

Prior to inputting the dataset into the classifiers, this study employed a widely recognized feature selection technique to identify the most crucial factors in predicting the prognosis of poisoned patients eligible for dialysis. At this stage, the relief feature selection algorithm was used to determine the importance or weight of each variable selected from the full-featured dataset. This algorithm operates with the same methodology as the K-NN algorithm, calculating the weight of each feature. The subsequent section presents the results of the executed feature selection algorithm, highlighting the selected variables and their respective ranks based on their weights (Table 2).

**Table 2:** Features chosen by the Relief algorithm and feature weight

Order	Feature name	Importance (feature weight, %)
1	Age	25.3
2	Intubation	24.07
3	PH	23.4
4	History of previous disease	18.5
5	HCO3	18.02
6	GCS	15.9
7	ICU Admission	11.71
8	Acute kidney injury	11.53
9	Potassium	9.66
10	Hypotension	9.49

The relief algorithm ranked ten key features, i.e., Age, Intubation, PH, history of previous disease, HCO3, GCS, ICU admission, Acute kidney injury, and potassium (Table 2). Based on this ranking, the top three predictive features for the prognosis of dialysis candidates who have been poisoned are age, intubation, and blood PH.

### k-fold cross-validation

The features were identified by the Relief Feature Selection algorithm and the complete dataset was tested on four ML models using a tenfold cross-validation methodology. This approach involved utilizing 90% of the poisoned patient dataset to train the ML models and reserving the remaining 10% to test the algorithms. To better evaluate the ML model's performance, this research calculated the average metric values from the tenfold cross-validation, supplemented with a 95% confidence interval. The best hyperparameters for each machine learning algorithm, summarized in Table 3, were selected based on this approach to maximize performance on the dataset.

**Table 3:** Best hyper parameters selected for machine learning algorithms

ML algorithms	Hyper parameters	Importance
XGB	‘min_chid_weigh’ = 4’max_depht’ = 12,’learning_rate’ = 0.4, ‘gamma’ = 0.6, ‘colsample_bytree’ = 0.9	0.88
K-NN	(leaf_size = list(range(1,20)), n_neighbors = list(range(1,9)), *p* = [1, 2])	0.73
AdaBoost	(“random_state”: 924, “n_estimators”: 92, “learning rate”: 0.4, “algorithm”: “samme.R”)	0.89
HGB	(‘verbose’ = 4, ‘random_state’ = 84, ‘max_leaf_nodes’ = 78, ‘max_iter’ = 180, ‘max_depht’ = 11, ‘learning rate’ = 0.8)	0.94

The results of tenfold cross-validation for four classifiers to predict the prognosis of poisoned patients undergoing hemodialysis are shown in Tables 4 and 5.

**Table 4:** Average evaluation metrics from ten runs of ML models

Classifier		Mean Accuracy	Mean Specificity (%)	Mean Sensitivity	Mean F- measure	ROC Rate
K-NN	Mean	0.738	0.718	0.796	0.74	0.784
	95% CI	(0.72, 0.75)	(0.69, 0.72)	(0.78, 0.81)	(0.72, 0.76)	(0.78, 0.79)
	STD	0.0314	0.014	0.021	0.0381	0.014
XGB	Mean	0.88	0.841	0.87	0.835	0.89
	95% CI^a^	(0.87, 0.89)	(0.83, 0.85)	(0.85, 0.89)	(0.83, 0.84)	(0.87, 0.9)
	STD^a^	0.03	0.027	0.029	0.001	0.014

^a^*CI* Confidence interval, *STD* Standard deviation

**Table 5:** Average evaluation metrics obtained from ten runs of ML models

Classifier		Mean Accuracy	Mean Specificity (%)	Mean Sensitivity	Mean F- measure	ROC Rate
AdaBoost	Mean	0.89	0.86	0.91	0.91	0.927
	95% CI^a^	(0.89, 0.91)	(0.85, 0.87)	(0.89, 0.93)	(0.87, 0.93)	(0.91, 0.92)
	STD^a^	0.002	0.0014	0.002	0.004	0.0018
HGB	Mean	0.948	0.935	0.94	0.892	0.92
	95% CI	(0.93, 0.96)	(0.92, 0.94)	(0. 93, 0.96)	(0.88, 0. 91)	(0.91, 0.93)
	STD	0.029	0.003	0.0125	0.019	0.010

^a^*CI* Confidence interval, *STD* Standard deviation

**Fig. 3:**
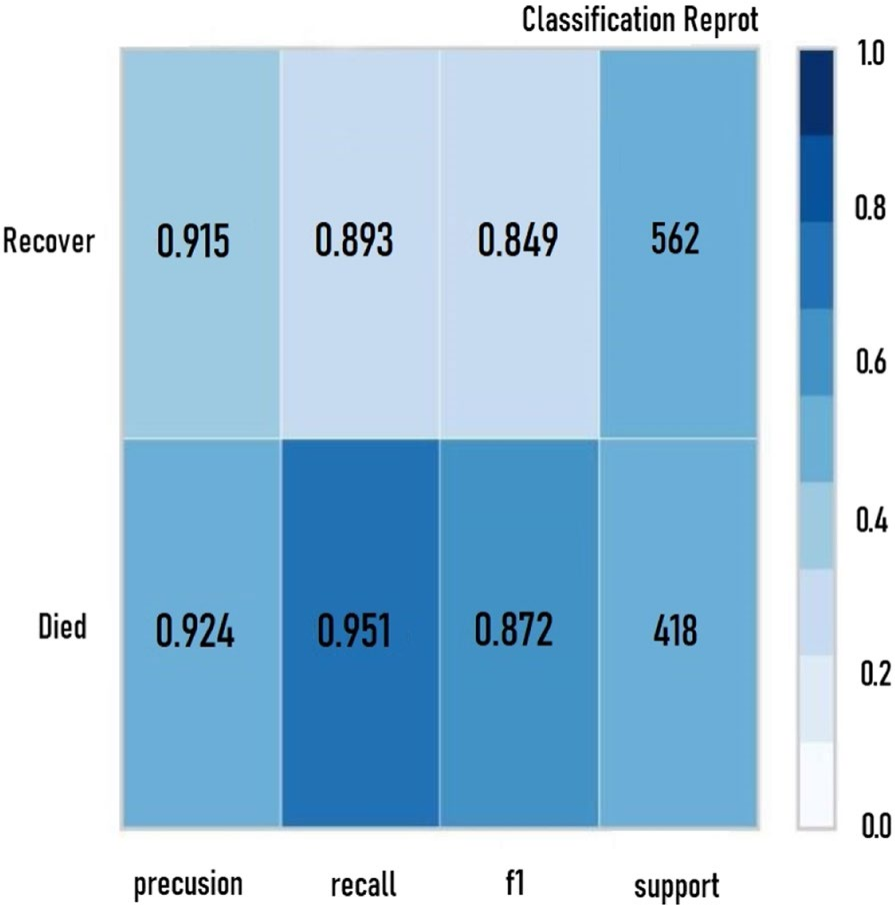
Classification report for HGB classifier

Figure 3 showcases a classification report diagram for the top-performing ML model, chosen according to the highest evaluation metrics and the optimal AUC rate. The time taken to construct each model, measured in seconds, and the Kappa Statistic (KS) metrics for all four classifiers are detailed in Table 6.

**Table 6:** Building time, training, and testing errors of selected classifiers

Evaluation criteria	Classifier			
	K-NN	AdaBoost	XGB	HGB
Best building time model (s)	174	9 7	61	75
Kappa statistic	81%	87.10%	90%	91.24%

The data presented in Tables 3 and 5 indicates that the HGB classifier outperformed the other three models when tested on the dataset. It achieved an average classification accuracy of 94.8%, a specificity of 93.5%, a sensitivity of 94%, an F-score of 89.2%, and a ROC of 92%. The time taken to execute the machine learning algorithms are showed in Table 6.

As shown in Table 6, the XGB algorithm outperforms others in terms of speed, requiring merely 61 s for model construction. The HGB Classifier is the second fastest, requiring approximately 75 s to build the prediction model. On the other hand, the K-NN model takes up to 174 s for model building, while the AdaBoost algorithm takes 97 s. Figure 3 illustrates the error rates of classifiers when applied to the given dataset.

The Kappa metrics and error rates of classifiers indicated that the XGB algorithm and the HGB classifier perform the best, with Kappa metrics rates of 90% and 91%, respectively.

The results demonstrated that the HGB classifier outperforms the other classifiers in this study, as indicated by its superior Kappa metrics and lower error rate. Thus, given these findings, the HGB classifier is considered the optimal algorithm for building a CDSS interface to predict the prognosis of poisoned patients who may need dialysis. Figure 3 showcases the classification report for the HGB classifier, highlighting it as the highest-performing ML model in this study based on the assessment metrics.

## Discussion

The present study aimed to forecast the prognosis of patients who have been poisoned and qualify for dialysis, utilizing innovative machine learning methods. As per the algorithm outcomes, the most crucial predictors of hemodialysis treatment results were age, intubation, PH, history of previous diseases, HCO3, GCS, ICU admission, acute kidney injury, potassium, and hypotension. Among the four models that were assessed, the HGB classifier demonstrated exceptional performance on the test dataset. It attained an impressive average accuracy rate of 94.8%, an average specificity rate of 93.5%, an average sensitivity rate of 94%, an average F-score of 89.2%, and an average ROC of 92%.

Several studies have reported influencing factors on the prognosis of poisoned patients who are candidates for dialysis. In their study of methanol poisoning cases treated with hemodialysis, Pajouhmand et al. uncovered a significant connection between the length of dialysis and the presence of dyspnea (*P* = 0.028) and the quantity of alcohol ingested (*P* = 0.02). Following a logistic regression analysis, the only statistically significant difference between the groups was creatinine levels (*P* = 0.02) [35]. In their study, Soucie et al. examined the factors that increase the likelihood of death among patients undergoing dialysis within the first 90 days. They discovered that advanced age, being of white race and male gender, experiencing physical and nutritional limitations, smoking, and having a history of cancer, congestive heart failure, clinical depression, or myocardial infarction all contribute to an elevated risk of mortality in individuals [36]. According to Msaad et al., hemodialysis patients with cardiovascular diseases, undernutrition, and inflammation have a higher mortality risk [37]. Kute et al. conducted a study on hemodialysis patients who were poisoned by methanol and found that those with severe metabolic acidosis (pH ≤ 6.90), requiring a ventilator, and experiencing coma/seizures upon admission had a higher incidence of death [38]. The studies indicate that the factors obtained in each study are influenced by the cause of hemodialysis or dialysis in patients and the population being studied. Our study, which comprehensively examined all poisoning factors leading to hemodialysis, revealed that factors such as age, intubation, previous disease history, and blood pH are similar to those found in other studies. There have been no prior investigations conducted using artificial intelligence on this specific sample model, which comprises poisoned dialysis patients.

Montemayor et al. research demonstrated that random forest is a more effective method than logistic regression for developing mortality prediction models in hemodialysis patients. The random forest model had a significantly higher AUC compared to the logistic regression model (with a difference of 3.78% and a *p*-value of less than 0.001) [19]. The observations of Radović et al. showed that the expected mortality rate for hemodialysis patients was determined using the Kernel support vector machine algorithm and K-means clustering algorithm. The accuracy of mortality rate prediction was up to 94.12% and up to 96.77% when observing a full database or a reduced database containing data for three major diseases [39]. Ahmed I. Akl conducted a study where a neural network (NN) model and direct dialysate quantification (DDQ) were utilized to predict urea concentrations during hemodialysis. The results of the NN model were compared to the direct dialysate quantification (DDQ) model, and the prediction error was found to be 10.9%. The researchers determined that using artificial intelligence in urea kinetics can provide insight into intradialysis profiling based on each person's specific clinical requirements [40].

A study conducted by Jiao Hu et al. focused on predicting the serum albumin level in hemodialysis patients. To achieve this, they utilized an enhanced version of the binary mutant quantum grey wolf optimizer (MQGWO) in combination with the fuzzy k-nearest neighbor (FKNN). The results showed an impressive accuracy of 98.39% and a specificity of 96.77%. These findings suggest that this model holds significant promise for identifying trends in serum albumin levels among HD patients [41]. In our study, the HGB classifier showed better performance on the test dataset. The effectiveness of a machine learning model, like the HGB classifier, compared to other classifiers, varies depending on the particular problem and dataset at hand. By comparing the performance of various machine learning algorithms for different tasks, we can gain a better understanding of their relative strengths and weaknesses [42]. There are two primary benefits to using HGB, which are the ability to handle missing data and its flexibility and scalability. HGB has the capability to handle missing values, making it useful in situations where data may be incomplete. Additionally, HGB is highly flexible and scalable, allowing it to be applied in a variety of different applications [43].

### Limitation

Since we used a retrospective dataset, there were certain fields that were missing or contained noisy information (such as incoherent, incomplete, abnormal, meaningless, and erroneous data), which could have affected the modeling process. To address this, we sought the expertise of two clinical toxicologists to define the normal range for each variable. Any values that fell outside this range were identified and completed by referring to patient records or consulting the responsible physician. Additionally, records with more than 70% empty fields were removed and imputed with mean or mode values for continuous and discrete variables, respectively.

The dataset did not include data on economic status, lifestyle habits, molecular biology, genomic or proteomic factors that could potentially impact the prediction of poisoned patients. Including these factors may enhance the predictive power of the models. Therefore, it is recommended that further studies be conducted with more accurate validations to improve the quality of modeling and minimize any bias in prognosis.

## Conclusion

This research is the first to use ML models for predicting the outcome of poisoned patients who are about to undergo hemodialysis. The most effective model for prognosis prediction was found to be HGB. This system can be utilized by physicians and clinical toxicologists to guide their interventions in order to reduce mortality and minimize the consequences of hemodialysis on poisoned patients, thus alleviating the strain on the healthcare system.

## Data Availability

Please contact the corresponding author with reasonable inquiries for data used or analyzed in this research.

## Abbreviations

ML: Machine learning
XGBoost: Extreme Gradient Boosting
HGB: HistGradient Boosting
KNN: K-Nearest Neighbors
AdaBoost: Adaptive Boosting
HCO3: Bicarbonate
GCS: Glasgow Coma Scale
ICU: Intensive care unit
WHO: The World Health Organization
CHP: Charcoal Hemoperfusion
CVVH: Continuous Venovenous Hemofiltration
CVVHDF: Continuous Venovenous Hemodiafiltration
BUN: Blood urea nitrogen
WBC: White blood cells
CDSS: Clinical Decision Support System
CV: Cross-validation
NN: Neural network
DDQ: Direct dialysate quantification
MQGWO: Mutant quantum grey wolf optimizer
FKNN: Fuzzy k-nearest neighbor
